# Global patterns and trends in spontaneous reporting systems: VigiBase, FAERS, VAERS, and JADER

**DOI:** 10.3389/fdsfr.2026.1919822

**Published:** 2026-09-04

**Authors:** Nikola Panajotovikj, Jordi Mestres

**Affiliations:** 1 Chemotargets SL, Parc Cientific de Barcelona, Barcelona, Spain; 2 Institut de Quimica Computacional i Catalisi, Universitat de Girona, Girona, Spain

**Keywords:** FAERS, JADER, pharmacovigilance, signal detection, VAERS, VigiBase

## Abstract

**Introduction:**

Spontaneous reporting systems (SRSs) gather millions of reports of adverse events occurring to patients worldwide. For its volume and breadth of data, they are often regarded as the cornerstone of pharmacovigilance. However, their individual and intra-database characteristics remain incompletely described.

**Methods:**

Four SRSs were considered, namely, the WHO global database VigiBase, the FDA Adverse Event Reporting System (FAERS), the Vaccine Adverse Event Reporting System (VAERS), and the Japanese Adverse Drug Event Report database (JADER). As of 31 December 2025, these databases contain approximately 42.4 million, 26.3 million, 2.7 million and 0.9 million reports, respectively. Following deduplication, approximately 11.1 million reports were uniquely shared between VigiBase and FAERS and close to 1.3 million between VigiBase and VAERS.

**Results:**

We described and compared report features such as drug and adverse event reporting patterns, reporter types, geographic distribution, demographics, and medical outcomes. Most reports have been received in recent years across the four databases with up to 70% of the reports accumulated in the last 7–14 years, depending on the SRS. Notable patterns appeared in reporter types (consumer-dominated in FAERS versus physician-dominated in JADER), sex (female predominance in VigiBase, FAERS and VAERS, versus near-parity in JADER), and the influence of COVID-19 vaccination campaigns, which disproportionately affected VigiBase and VAERS. Time-series trends were analysed by comparing reports pre and post 1 January 2015, excluding COVID-19 vaccine reports. Changes in reporting patterns of therapeutic subgroups and adverse events since 2015 were consistent across SRSs with some region-specific variations.

**Discussion:**

By highlighting differences between SRSs, this manuscript serves as a reference for researchers and regulators.

## 1 Introduction

Spontaneous reporting systems (SRSs) collect individual case safety reports (ICSRs) submitted by healthcare professionals, consumers, and marketing authorisation holders (MAHs). In turn, these data are used for detection of drug safety signals and regulatory decision-making (Jiao et al., 2024; Kim et al., 2024; Lomeli-Silva et al., 2024).

There are many SRSs operating worldwide. The Uppsala Monitoring Centre (UMC), on behalf of the World Health Organization (WHO), maintains VigiBase, which is the largest global system collecting post-marketing safety reports from more than 160 member countries of the WHO Programme for International Drug Monitoring (PIDM) (Lindquist, 2008; Potter et al., 2025). In the United States there are two principal SRSs: the Food and Drug Administration (FDA) Adverse Event Reporting System (FAERS), mainly collecting medicine-related adverse event reports (openFDA), and the Vaccine Adverse Event Reporting System (VAERS) co-managed by the FDA and the Centers for Disease Control and Prevention, focusing on vaccine-related reports (CDC, 2026; VAERS - Data, 2026). On the other hand, Japan’s Pharmaceuticals and Medical Devices Agency (PMDA) manages the Japanese Adverse Drug Event Report database (JADER), which captures reports within the Japanese regulatory framework (Tsuchiya et al., 2025; Pharmaceuticals and Medical Devices Agency, 2026).

Previous publications have individually characterised these SRSs, however comparisons between databases remain scarce (Liu et al., 2024; Dedefo et al., 2025). It’s crucial for researchers and regulators who conduct multi-database pharmacovigilance studies to understand the similarities and differences across SRSs. Changes in reporting patterns, regulatory frameworks, geographic coverage, and data collection strategies can influence the characteristics of collected reports and possibly affect the detection of safety signals and their interpretation (Mestres, 2024).

Brand et al. recently described the patterns and trends in adverse event reporting in VigiBase, providing an updated profile overview of the database (Brand et al., 2026). The present study aims to extend their work by performing a systematic comparison across VigiBase, FAERS, VAERS and JADER. Specifically, we intend to: (i) quantify the shared reports between databases using a deduplication algorithm; (ii) characterise and compare reporting patterns as of 31 December 2025; (iii) examine temporal trends by comparing reports pre and post 1 January 2015; and (iv) describe shifts in reporting of therapeutic subgroup and adverse event category. Additionally, we examined the impact of the COVID-19 vaccination campaigns on reporting volumes across the four SRSs, building on previous observations (Montes-Grajales et al., 2023; Brand et al., 2026). This study follows a descriptive, cross-sectional design and its primary aim is to characterise and compare reporting patterns across databases. Accordingly, all observed differences should be interpreted as reflecting characteristics of the reporting systems rather than underlying clinical or epidemiological differences.

## 2 Materials and methods

### 2.1 Data sources

All data used in this work were extracted from the four major SRSs as of 31 December 2025. VigiBase (Lindquist, 2008; Potter et al., 2025), as an aggregator database that collects reports from all around the world, is the largest SRS, containing 42.4 million reports. FAERS (openFDA), VAERS (CDC, 2026; VAERS - Data, 2026), and JADER (Tsuchiya et al., 2025; Pharmaceuticals and Medical Devices Agency, 2026) contain 26.3, 2.7, and 0.9 million spontaneous reports, respectively.

### 2.2 Data processing

The identification of both intra- and inter-source duplicate reports was based on content matching of the variables “drug codes” (ICH E2B fields G.k.2.1.1b, G.k.2.1.2b, G.k.2.3. r.2 b), “adverse event” (E.i.1.1a, E. i.1.1b, E. i.1.2, E. i.2.1a, E. i.2.1 b), “time to onset mean” (G.k.9. i.3.1 b), “indication” (G.k.7. r.1), “basis” (G.k.1), “event day” (E.i.4), “sex” (D.5), “region” (derived from the country code, C.2. r.3), and “age” (D.2.2 b), with a previous transformation step to make contents comparable in terms of units or ranges. Drugs were harmonised using a curated drug dictionary, which includes the WHO Drug Dictionary and the ATC classification, and adverse events were matched at the MedDRA™ Preferred Term (PT) level.

Duplicates were then classified by how closely two reports agreed, following the scheme of Montes-Grajales et al. (2023). Three types were distinguished: (a) an exact match on every compared field; (b) a single missing element in exactly one of “adverse event”, “basis”, or “indication”; and (c) a single-character difference (Levenshtein distance = 1) in exactly one of those same three fields, with exact matches on all others. For the “adverse events” field specifically, Type b describes two reports that agree in every other respect but differ by the presence or absence of one entry in the multi-valued event list. Allowing these near-matches (Types b and c) ensured that pairs separated only by data-entry variation, rather than by genuinely different events, were still recognised as duplicates. Comparisons were restricted to fields populated in both reports, and a field absent from both was treated as a match. The time-to-onset mean across all drug-event combinations within a report was used as a summary variable for duplicate detection, as matching on individual combination-level values would multiply the comparison space and reduce matching precision. Like any content-based approach, this scheme can produce errors in two directions: over-deduplication, which discards genuine reports, and under-deduplication, which leaves residual overlap between sources.

The Jaccard similarity index between two databases at a given time point was calculated as the number of shared reports divided by the total number of reports in their union, providing a measure of report overlap over time.

### 2.3 Data stratification

We collected descriptive statistics across all four SRSs, for the following report variables: geographic location, reporter type, patient demographics (age, sex, weight), drug and adverse event count, medical outcomes, and therapeutic indications. To facilitate cross-database comparison, only reports with non-missing values for those variables were considered in the statistical analyses. Variable contents were stratified in different groups as follows:

Geographic location. Reports were classified by geographic region according to the six WHO regional groupings: African Region, Eastern Mediterranean Region, European Region, Region of the Americas, South-East Asia Region, and Western Pacific Region. Country-level information, where available, was mapped to the corresponding WHO region.

Reporter type. Reporters were categorised into five groups: consumer, physician, pharmacist, other healthcare professional (OHP), and lawyer. Because VAERS does not provide reporter type in a standardised structured form directly comparable to the other three databases, reporter type analysis was limited to VigiBase, FAERS, and JADER.

Patient age. Age values in reports were binned into eight groups: newborn (≤27 days), infancy (28 days to <2 years), childhood (2–11 years), adolescence (12–17 years), adulthood (18–44 years), midlife (45–64 years), maturity (65–74 years), and retirement (≥75 years). When age was reported in units other than years (e.g., days, months), it was converted to years before binning.

Patient sex. Sex was classified as female or male. Reports with missing, unknown, or indeterminate sex were excluded from sex-specific analyses.

Patient weight. To enable a standardised comparison of weight distributions across databases, we estimated the body mass index (BMI) using the reported patient weight and population-level average height values for each sex. Specifically, the average adult male height (170.7 cm) and average adult female height (158.6 cm) were used as fixed height proxies. BMI was then calculated as weight (kg) divided by the square of the estimated height (m^2^) and classified according to the WHO categories: underweight (<18.5 kg/m^2^), normal weight (18.5–24.9 kg/m^2^), overweight (25.0–29.9 kg/m^2^), and obese (≥30.0 kg/m^2^). This approach provides only an approximate BMI classification and should be interpreted as exploratory, the use of fixed average heights introduces systematic error, underestimating BMI for shorter individuals and overestimating it for taller ones. VAERS does not capture patient weight and was therefore excluded from weight analyses.

Medical outcome. Criteria of medical outcome seriousness were analysed as reported in each database. Categories included death, life-threatening events, hospitalisation, disability, and other serious medical events, although the specific categories available varied across databases.

Therapeutic indication. The number of reported indications per report was assessed and the most frequently reported indications were identified. Because VAERS reports vaccines, which are generally administered with the single indication of immunisation, indication analysis was limited to VigiBase, FAERS, and JADER.

Drug reporting. The total number of drugs listed per report was counted, including both suspected and concomitant medications. Reports were grouped in three categories based on the number of drugs listed: 1, 2–5, and 6 or more. The number of drugs flagged as suspected of causing the adverse event was counted separately and grouped using the same categories as above.

Adverse event reporting. The number of adverse event terms (PTs) listed per report was counted and grouped into three categories: 1, 2–5, and 6 or more. Every adverse event mentioned in a drug report is considered as a drug-event pair (DEP).

Temporal trends. Trends over time were examined by comparing report characteristics before and after 1 January 2015, following the analytical framework previously established for VigiBase (Brand et al., 2026), and extending it to FAERS, VAERS, and JADER. This date was selected to maintain consistency with the framework of Brand et al. (2026). COVID-19 vaccine reports were excluded to avoid obscuring underlying trends (Montes-Grajales et al., 2023). A COVID-19 vaccine report is any report that mentions a COVID-19 vaccine, regardless of whether other therapeutics are reported alongside it or not. Shifts in the distribution of suspected medicinal products by Anatomical Therapeutic Chemical (ATC) therapeutic subgroup and adverse events by MedDRA® High Level Group Term (HLGT) were analysed overall and stratified by geographic area and reporter type.

### 2.4 Signals of disproportionate reporting

The reporting disproportionality of an adverse event for a drug was quantified by calculating the proportional reporting ratio (PRR). For each DEP, the PRR and its 95% confidence interval (PRR05-PRR95) were calculated cumulatively at each year, from the year of the first deposited report for the drug through 2025. Following previous works (Slattery el al., 2013; Cutroneo et al., 2024), a DEP was labelled as a signal of disproportionate reporting (SDR) at a given year if the lower bound of the 95% confidence interval of the PRR exceeded 1 (PRR05 > 1) and the pair was supported by at least five reports. Reports containing COVID-19 vaccines were excluded from all SDR analyses. SDR yield was defined as the number of SDRs divided by the number of DEPs for a database multiplied by 100. SDR yield is a descriptive metric reflecting a database’s propensity to generate statistical signals; differences in reporting culture, product mix, database scope, and report quality all contribute to the observed variation.

To characterise reporting within each database, we quantified reporting bias using Shannon entropy. For a database containing N unique categories (PTs or DEPs), each with report count c_i_, the proportion of reports attributed to category i is p_i_ = c_i_/Σc_i_. Shannon entropy was calculated as E = −Σ p_i_ log_2_ (p_i_) and normalised by the maximum entropy for N categories (E_max_ = log_2_(N)) to yield a value between 0 and 1, where 1 indicates a perfectly uniform distribution and lower values indicate greater concentration of reports in fewer categories. This analysis was performed at two levels: across MedDRA™ PTs of adverse drug events (ADE) alone (ADE entropy) and across unique DEP combinations (DEP entropy).

## 3 Results and discussion

Not all report variables are directly comparable across the four systems. Where a variable is unavailable in a given database, that database is excluded from the corresponding analysis, as indicated in each subsection.

### 3.1 Database overview

**FIGURE 1:**
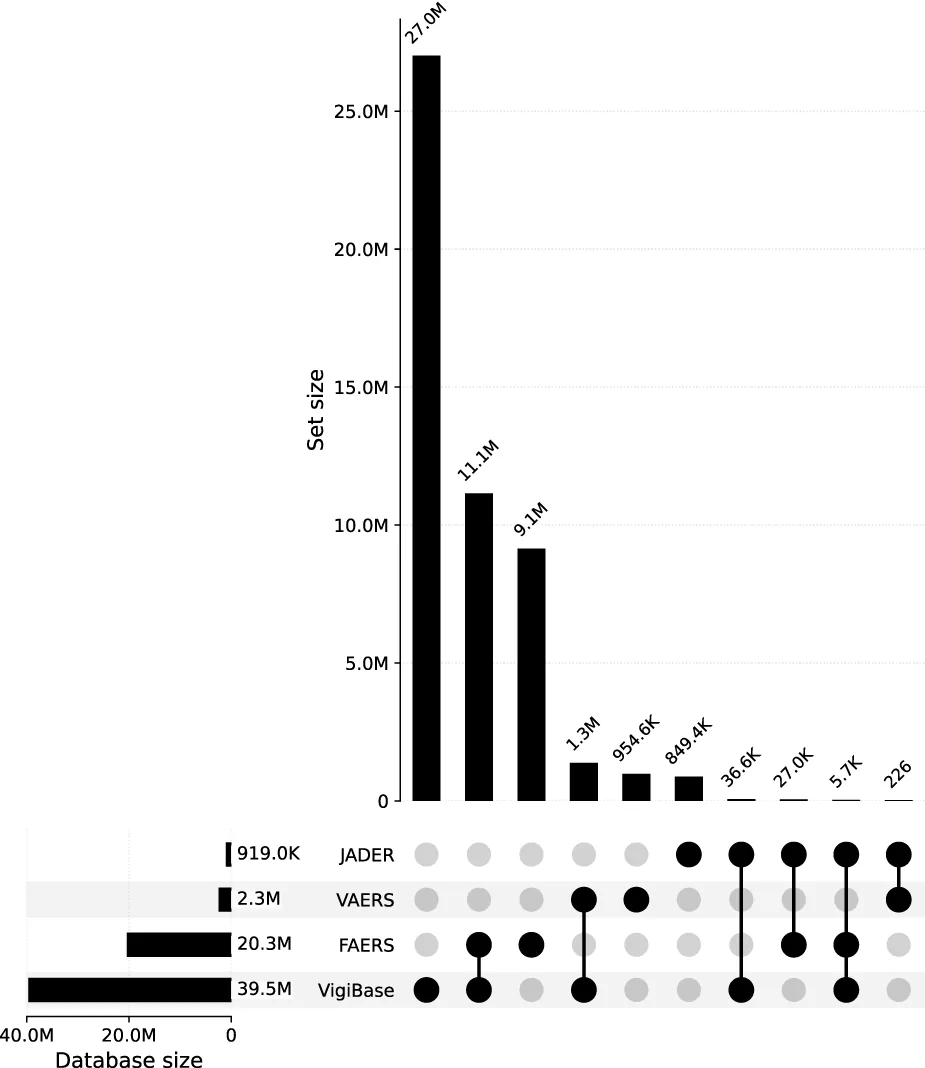
Total number of deduplicated reports per database as of 31 December 2025.

Database content and report overlap. As illustrated in Figure 1, following deduplication VigiBase remains the largest SRS with 39.5 million reports, of which 27.0 million (68.4%) are exclusive to the source. FAERS follows next in size with 20.3 million reports, 9.1 million (44.8%) of which are exclusive. VAERS is left with 2.3 million, 1.0 million (41.5%) exclusive, and JADER retains most of its 0.9 million reports, the large majority of them (92.4%) being exclusive. The deduplication analysis reveals also considerable overlap between databases, with VigiBase incorporating more than half of the entire contents of both FAERS (54.7%) and VAERS (56.5%). This emphasizes the importance of accounting for inter-source duplicates when performing disproportionality analyses with integrated SRSs.

A total of 8,485 drugs were detected in the final set of unique reports. Of those, around 3,343 were uniquely shared between VigiBase and FAERS, 1,689 between VigiBase, FAERS and VAERS, and 1,696 between all four SRSs. This accounts for 6,728 drugs (79.29%), the rest are drugs that are reported in lower numbers between combinations of SRSs or individual systems (Supplementary Figure S1A). Particular attention was put into identifying reports containing vaccines exclusively from reports containing any other therapeutic modality, with or without vaccines (Supplementary Table S1; Supplementary Table S2). This revealed that 19.3% of all reports in VigiBase contain only vaccines, whereas JADER and FAERS are composed of 5.4% and 1.6% of vaccine-only reports, respectively. VAERS, designed to be a vaccine database, predominantly (89.3%) contains reports of vaccines exclusively.

On the other hand, the total number of reported MedDRA® PTs detected in all SRSs was 24,537. Interestingly, 38.7% of them (9,496) are shared across all four SRSs, and only 4.0% (985) are exclusively reported in an individual SRS (Supplementary Figure S1B).

Impact of COVID-19 vaccination campaigns. The global vaccination campaigns as a response to the COVID-19 pandemic had a substantial impact on healthcare and reporting of adverse events. This is reflected in the SRSs that accept vaccine reports. For instance, 13.9% and 57.8% of all reports in VigiBase and VAERS, respectively, are related to a COVID-19 vaccine (Supplementary Figure S2). This represents a significant influx of data in a relatively short period of time that altered the overall composition of the databases and affected safety signal detection in these SRSs (Montes-Grajales et al., 2023). Conversely, FAERS remains mostly unaffected (with only 0.3% of COVID-19 vaccine reports) because of the US regulatory framework redirecting vaccine reports to VAERS. In JADER, COVID-19 vaccine reports make up 3.7% of its total volume. These findings are consistent with previous observations (Montes-Grajales et al., 2023; Brand et al., 2026) highlighting the impact of pandemic-related reporting across SRSs with different scopes and regulatory mandates.

**FIGURE 2:**
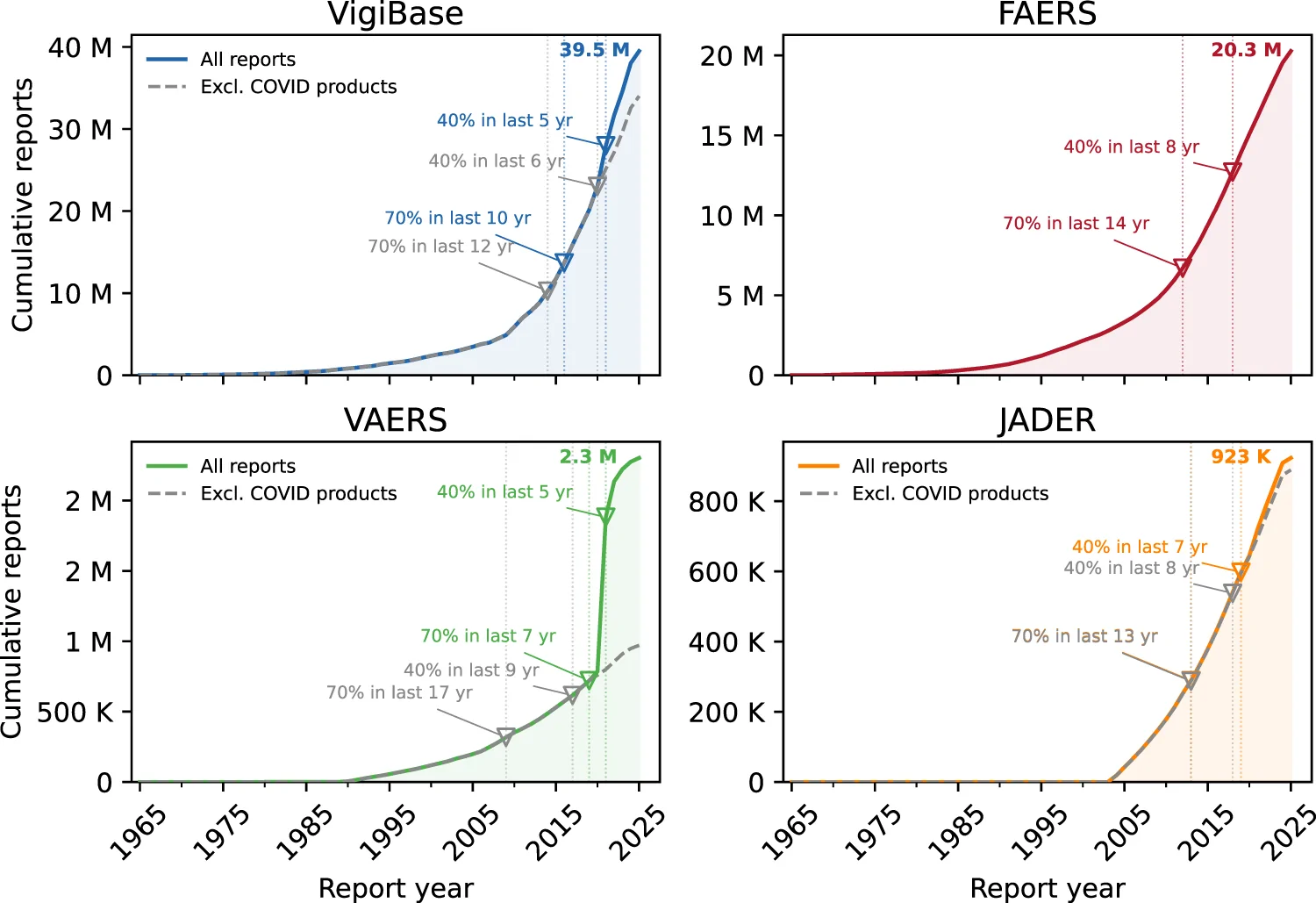
Cumulative reports by calendar year for VigiBase, FAERS, VAERS and JADER.

Report Accumulation Over Time. Figure 2 summarises the annual accumulation of reports across the four databases and shows consistent accelerated growth across all of them. For example, consistent with a recent analysis (Brand et al., 2026), approximately 40% and 70% of all reports in VigiBase were received in the past 5 (2021) and 10 (2016) years, respectively. In comparison, FAERS collected 40% and 70% of all its reports in the last 8 (2018) and 14 (2012) years, with a similar outcome observed for JADER. The massive entry of reports in VAERS due to the COVID-19 pandemic is reflected in the shortest period (7 years, 2019) to collect 70% of all vaccine reports (Figure 2).

This accelerated growth is consistent with several factors, such as increased regulatory requirements, expansion of electronic reporting systems and greater patient involvement in pharmacovigilance, but also intensified reporting activity during the COVID-19 pandemic. Regarding the latter, removal of reports containing COVID-19 vaccines extends the time to attain 40% and 70% of all SRS contents, except FAERS. For example, exclusion of COVID-19 reports extends 1 year the accumulation of 40% of all reports in VigiBase (from 5 to 6 years) and JADER (from 7 to 8 years). Naturally, VAERS experiences the strongest impact of COVID-19 in its growth, with a delay of 4 years (from 5 to 9 years) and 10 years (from 7 to 17 years) the deposition of the last 40% and 70% of all reports, respectively (Figure 2).

### 3.2 Key report characteristics

This section presents a comprehensive summary of report characteristics across the four databases as of 31 December 2025. All reports containing COVID-19 vaccines have been removed as their overwhelming number could bias the reporting patterns observed. The values discussed below are summarised in Supplementary Table S3.

**FIGURE 3:**
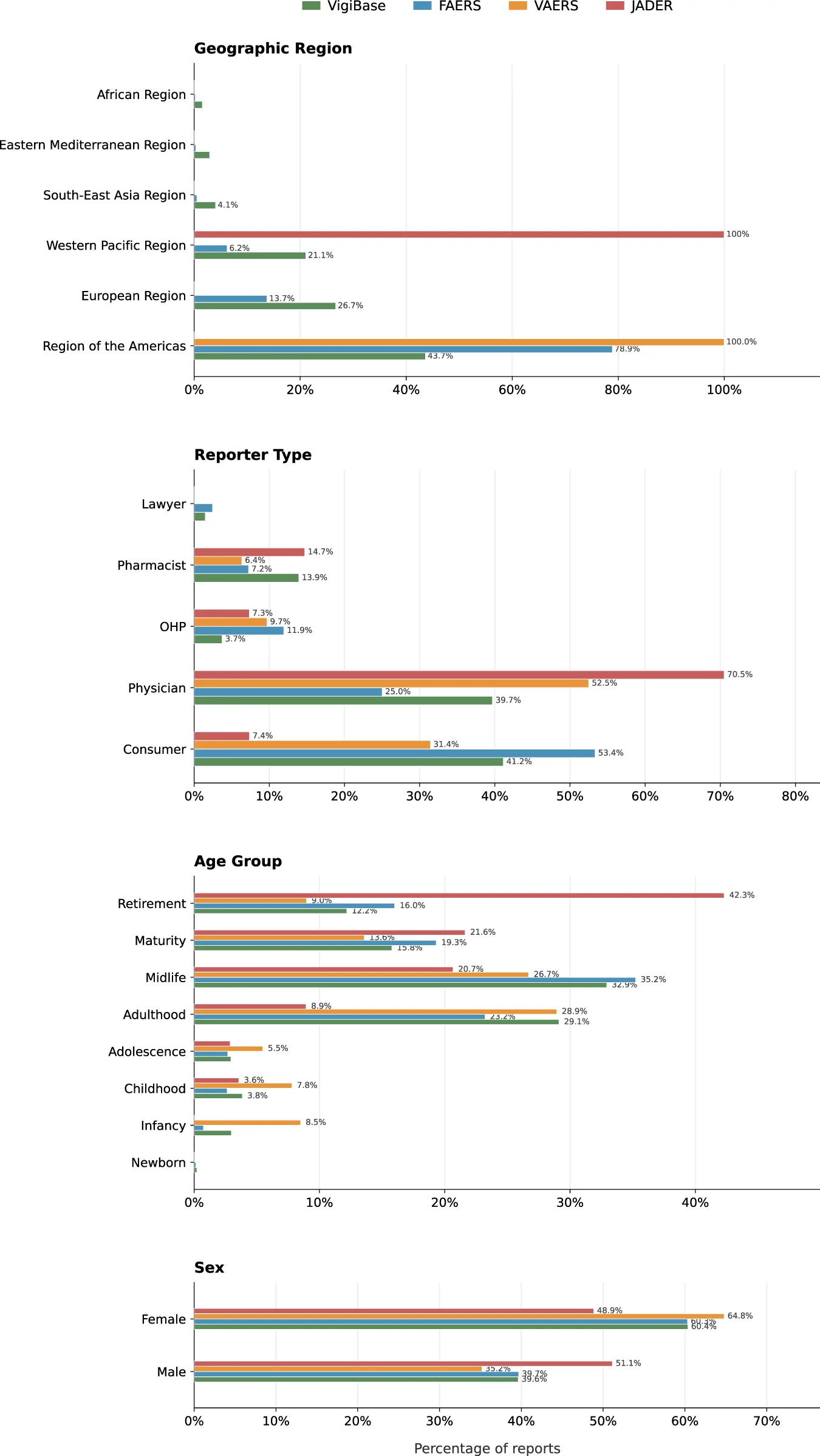
Distribution of key report characteristics across VigiBase, FAERS, VAERS, and JADER after exclusion of COVID-19 vaccine reports. Shown are the proportions of reports by geographic region, reporter type, age group, and sex, calculated among reports with non-missing values for each variable. Age groups are defined as follows: Newborn (≤27 d), Infancy (28 d-<2 y), Childhood (2–11 y), Adolescence (12–17 y), Adulthood (18–44 y), Midlife (45–64 y), Maturity (65–74 y), Retirement (≥75 y).

Geographic location. Geographic reporting patterns reflect the scope and regulatory context of each SRS (Figure 3). VigiBase exhibits the broadest geographic distribution, with reports from the Region of the Americas (43.7%), the European Region (26.7%), and the Western Pacific Region (21.1%), as well as minor contributions (<5%) from South-East Asia Region (4.1%), the Eastern Mediterranean Region (2.9%), and the African Region (1.6%), in good agreement with a recent review (Brand et al., 2026). FAERS predominantly contains reports from the Region of the Americas (78.9%), with significantly smaller contributions from the European Region (13.8%) and the Western Pacific Region (6.2%). VAERS and JADER contain reports exclusively from the Region of the Americas and the Western Pacific Region (Japan, in particular), respectively. Geographic region data are available for most reports across all databases (64.96%–100%), with VAERS having the lowest availability.

Reporter type. The distribution of reporter types varies considerably across databases (Figure 3). A balanced distribution between consumers (41.2%) and physicians (39.7%) is observed in VigiBase, with pharmacists representing 13.9%. However, in FAERS consumers constitute the largest reporter group (53.4%), followed by physicians (25.0%), OHP (11.9%), and pharmacists (7.2%). In contrast, in JADER physicians are the dominant reporters (70.5%), followed by pharmacists (14.7%) and consumers (7.4%), potentially reflecting the Japanese pharmacovigilance framework that mainly relies on adverse event reporting from healthcare professionals submitted to the PMDA (Liu and Wu, 2026). VAERS does not provide reporter type information in a standardised structured form directly comparable to the other databases (VAERS Data Use Guide, 2025). Spontaneous reports from lawyers are rare across all databases but, interestingly, they are most prominent in FAERS (2.5%).

Patient age. Age information is available in 76.6% of VigiBase reports, 60.4% in FAERS, 80.2% in VAERS, and 90.8% in JADER. In VigiBase and FAERS (Figure 3), the age distribution is centred on adults, with the midlife group (45–64 years) being the largest category (32.9%and 35.2%, respectively). VAERS shows a more even distribution across adult age groups, with a clear higher proportion of paediatric reports (infancy 8.5%, childhood 7.8%) consistent with the concentration of routine immunizations in infancy and childhood in the United States (Zauche et al., 2026). JADER markedly contains reports from older age profiles, with 42.3% of them in the retirement category (≥75 years), a pattern consistent with Japan’s ageing population and the associated burden of polypharmacy and age-related adverse events (Katsuno et al., 2021; Otani et al., 2024; Fujita et al., 2026).

Patient sex. Reports from female patients are clearly over-represented across VigiBase, FAERS, and VAERS at 60.4%, 60.3% and 64.8% respectively (Figure 3). Several explanations have been proposed, including women receiving more prescriptions, engaging more with healthcare systems, and/or being more susceptible to some adverse events (Lacroix et al., 2023; Sportiello and Capuano, 2024; Brand et al., 2026). JADER is a notable exception, with an approximately equal sex distribution (48.9% female *versus* 51.1% male). This balance may be related to differences in Japanese prescribing, reporting culture, or population demographics (Tanaka et al., 2025; Fujita et al., 2026). Sex data completeness varies substantially across SRSs, with VAERS and JADER showing the highest reporting rates (94.7% and 95.7%, respectively) and FAERS having the lowest (53.8%).

Patient weight. Weight information is the least consistently reported patient characteristic across databases (Supplementary Table S3). It’s available in 5.8% of VigiBase reports, 21.6% of FAERS reports, and 37.2% of JADER reports. VAERS does not capture this information (VAERS Data Use Guide, 2025). Weight is a non-mandatory field and may be preferentially reported when considered clinically relevant (e.g., as a risk factor or confounder), introducing selection bias; the distributions below therefore reflect the reported subpopulation and may not represent the broader patient populations. Among reports with patient weight data, the weight group distribution differs significantly between Western and Japanese databases. In VigiBase and FAERS, a substantial proportion of patients are classified as obese (33.8% and 28.9%) or overweight (25.6% and 24.6%). In contrast, nearly half the patients in JADER are classified as underweight (49.2%) and the other half being mostly defined as normal weight (44.3%), with very few patients classified as obese (1.5%) or overweight (5.1%). These differences are consistent with known population-level weight differences between geographical locations (OECD/WHO, 2024; OECD, 2026) and they should be accounted for when performing safety signal detection and analysis.

Medical outcomes. The reporting of medical outcome seriousness varies considerably across SRSs, both in availability and distribution (Supplementary Table S3). JADER has the highest reporting rate (72.2%), followed by FAERS (60.7%), VigiBase (27.4%), and VAERS (8.0%). Hospitalisation is the most frequently reported medical outcome category in VigiBase (31.8%) and FAERS (29.6%), but it isn’t captured as a separate category in JADER. VAERS exhibits a distinctive pattern, with disability (45.8%) and life-threatening events (27.5%) being the two most reported categories. Notably, death is reported in 22.4% of all reports with medical outcome information in VAERS, 11.7% in FAERS, 10.5% in VigiBase, and 8.6% in JADER. These differences could be influenced by database-specific definitions, reporting requirements, and the spectrum of products captured. SRSs are subject to underreporting, so these proportions should not be taken as reflecting the true incidence of fatal outcomes. Less serious reactions are far less likely to be reported, so the proportion of deaths substantially overestimates their true frequency in clinical practice (Costa et al., 2023).

Therapeutic indications. Indication data is available in 60.2% of VigiBase reports, 81.9% of FAERS reports, and 91.2% of JADER reports. In VAERS, indication is not reported, as vaccines are generally administered with the single indication of immunization. Among reports with indication data, most list a single indication (64.4%–82.1%). However, JADER has a particularly high proportion of reports listing 2–5 indications (30.2%), consistent with greater polypharmacy in an ageing population (Katsuno et al., 2021; Otani et al., 2024; Fujita et al., 2026). Of mention is the fact that the proportion of reports listing “Product used for unknown indication” is highest in FAERS (27.6%), followed by VigiBase (17.4%) and JADER (8.8%), suggesting variability in the quality and completeness of indication reporting across systems.

Number of drugs per report. The number of total drugs listed per report differs markedly across databases. Reports listing a single drug are most common in VAERS (62.3%), VigiBase (57.0%), and FAERS (51.7%). In JADER, only 30.5% of reports list a single drug, with 28.2% listing six or more. This pattern in JADER is consistent with the higher prevalence of polypharmacy in Japanese clinical practice, particularly among elderly patients (Mabuchi et al., 2020; Otani et al., 2024). Similarly, the number of suspected drugs per report follows comparable patterns, with most reports listing a single suspected drug across all databases (67.0%–81.2%).

Number of adverse events per report. The number of adverse events (MedDRA® PTs) listed per report also varies across systems. JADER has the highest proportion of single-event reports (68.3%), followed by VigiBase 46.9%, FAERS (37.3%), and VAERS (18.3%). Conversely, VAERS has the highest proportion of reports listing six or more adverse events (25.9%), which could reflect the comprehensive symptom reporting associated with vaccine SRS reporting. These patterns could have implications for disproportionality analyses and signal detection methodologies, as the number of co-reported events can influence statistical measures.

### 3.3 Temporal trends in report characteristics

**FIGURE 4:**
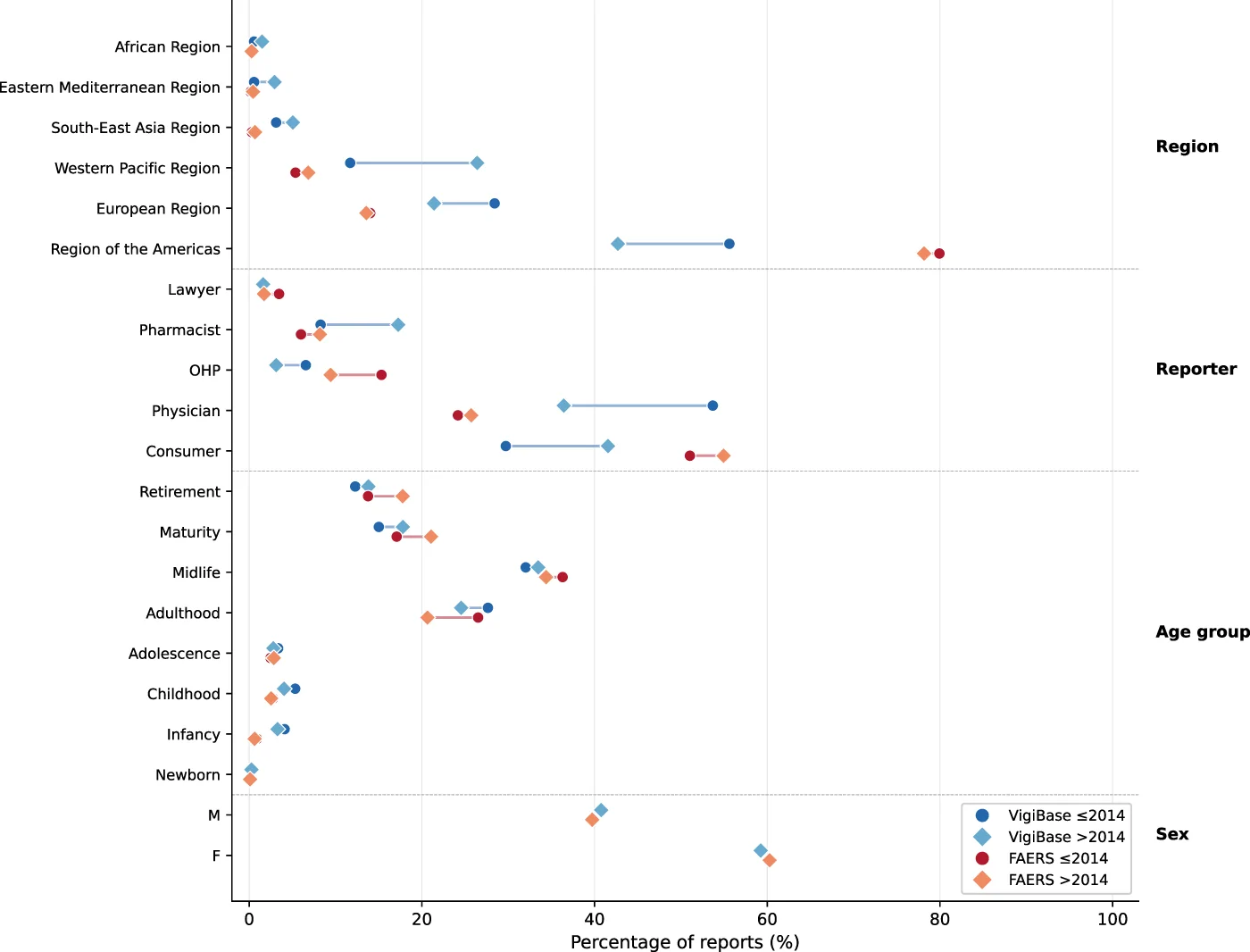
Changes in geographic region, reporter type, age group, and sex distributions before and after 1 January 2015 in VigiBase and FAERS, excluding COVID-19 vaccine reports. Points represent the percentage of reports with non-missing values for each variable for the two time periods. Age groups are defined as follows: Newborn (≤27 d), Infancy (28 d-<2 y), Childhood (2–11 y), Adolescence (12–17 y), Adulthood (18–44 y), Midlife (45–64 y), Maturity (65–74 y), Retirement (≥75 y).

The annual evolution of key report characteristics (geographic region, reporter type, patient sex, and age group) across all four databases, from the earliest available year for each SRS through 2025 and excluding COVID-19 vaccine reports, is presented in Supplementary Figure S3-S6. To identify changes in temporal trends in reporting patterns, we compared report characteristics for reports submitted before 1 January 2015 and from 2015 onwards following the analytical framework established by Brand et al. (2026) (Figure 4), excluding COVID-19 vaccine reports to avoid confounding the analysis. The values are summarised in Supplementary Table S4.

Geographic location. In VigiBase (Supplementary Figure S3), the strongest geographic shift is an increase in the proportion of reports from the Western Pacific Region (from 11.7% to 26.4%) and the Eastern Mediterranean Region (from 0.6% to 2.9%), with corresponding reduction in reports from the Region of the Americas (55.6%–42.7%) and the European Region (28.4%–21.4%) (Figure 4). This temporal pattern is consistent with the expansion of the WHO PIDM programme and increased pharmacovigilance efforts in these regions (Brand et al., 2026). In FAERS (Supplementary Figure S4), geographic distribution remains relatively stable, maintaining a high proportion of reports from the Region of the Americas (above 75% throughout) and with a slight increase in the proportion of reports from the Western Pacific Region (5.4%–6.9%). Contributions from the African Region and the Eastern Mediterranean Region increase modestly in both VigiBase and FAERS (Supplementary Figure S3 and S4). VAERS (Supplementary Figure S5) and JADER (Supplementary Figure S6) show no change in geographic distribution, consistent with their single-country scope (Supplementary Table S4).

Reporter types. A notable trend across databases is the increasing contribution of non-physician reporters (Figure 4). In VigiBase (Supplementary Figure S3), the proportion of reports from consumers increases from 29.7% to 41.6%, while physician-reported cases decline from 53.7% to 36.4%. Pharmacist reporting increases markedly (8.3%–17.3%). Similar trends are observed in FAERS (Supplementary Figure S4), where consumer reporting increases (51.0%–54.9%) and OHP reporting declines (15.3%–9.4%). In JADER (Supplementary Figure S6), physician-reported cases decrease substantially (82.0%–64.9%), with corresponding increases in consumer (3.3%–9.4%), OHP (4.0%–8.6%), and pharmacist (10.7%–17.1%) reporting. These trends are consistent across the four SRSs and may reflect changes promoting patient involvement and reporting in national pharmacovigilance systems (Cervantes-Arellano et al., 2024; Masiliūnienė et al., 2025; Brand et al., 2026; Tsuchiya et al., 2026).

Patient demographics. Across all SRSs, age reporting completeness generally declines after 2015, from 64.3% to 57.7% in FAERS, from 90.2% to 75.0% in VAERS, and from 94.9% to 88.3% in JADER (Figure 4). In contrast, age reporting in VigiBase remains relatively stable (75.7%–73.8%). However, a consistent shift towards older patient populations is observed across all databases, with increasing proportions of reports in the retirement and maturity categories and declining proportions in younger adult categories. In JADER, for example, the retirement category (≥75 years) increases from 32.3% in 2004 to 46.9% in 2025. This pattern is consistent with ageing populations, growing use of medicines in the elderly, and increased pharmacovigilance attention to this vulnerable group (McGettigan et al., 2024; Hart et al., 2025; Panajotovikj et al., 2025).

Sex distribution remains stable across SRSs (Figure 4), with the female predominance in FAERS, VAERS, and VigiBase, and the near parity in JADER persisting. Weight reporting declines in all databases that capture this variable, from 10.1% to 5.2% in VigiBase, from 25.5% to 18.8% in FAERS, and from 50.0% to 31.4% in JADER. Among reports containing patient weight data, a modest shift towards higher weight is observed in both VigiBase and FAERS.

Medical outcomes. The proportion of reports with medical outcome data declines in all databases after 2015. From the most to the least affected, a decrease from 82.0% to 66.7% is observed in JADER, from 68.8% to 55.0% in FAERS, from 32.1% to 27.5% in VigiBase, and from 5.9% to 4.6% in VAERS. Among those reports, while hospitalisation declines slightly in most databases, reports of death remain relatively stable across all systems.

Therapeutic indications. Indication reporting experiences a substantial increase in both VigiBase (from 51.2% to 68.9%) and FAERS (from 68.4% to 91.4%), possibly reflecting improved data collection practices and regulatory requirements. However, the proportion of reports listing “Product used for unknown indication” also increased markedly in VigiBase (11.1%–21.6%), FAERS (15.5%–34.0%), and JADER (2.0%–13.3%), suggesting that the increased capture of the indication data has not been accompanied by improvements in quality of indication data (Dedefo et al., 2025).

Drug and adverse event reporting patterns. Since 2015, there has been a trend towards listing fewer drugs per report across all databases, with increasing proportions of single-drug reports and decreasing proportions of reports with six or more drugs. This shift towards registering only one drug per report is more strongly apparent in JADER (from 21.14% to 33.36%) than in VigiBase (from 46.74% to 54.90%), FAERS (from 48.36% to 54.36%), and VAERS (from 25.69% to 28.36%). The number of adverse events per report remains relatively stable across databases and time periods.

Reporting completeness. VigiBase is the only SRS that provides a quality metric (vigiGrade) to assess the completeness of the information content provided in individual reports (Bergvall et al., 2013). Interestingly, the vigiGrade completeness score has remained relatively stable over the lifetime of VigiBase (Supplementary Figure S7). However, comparing reports submitted before and after 1 January 2015 reveals a modest improvement. The pre-2015 period has a mean vigiGrade score of 0.35, with a median (Q50) of 0.32, a first quartile (Q25) of 0.19, and a third quartile (Q75) of 0.48. In the post-2015 period, the mean vigiGrade increases slightly to 0.38, with a median (Q50) of 0.35, Q25 of 0.23, and Q75 of 0.52. This indicates that while overall completeness has improved marginally over time, the interquartile range has widened, suggesting greater variability in report quality in recent years. Reports from the most recent years (2023–2025) show a further increase in the upper quartile (Q75 reaching 0.60 in 2024 and 0.62 in 2025), potentially reflecting improved data collection practices in some contributing countries. Nonetheless, the relatively low median scores across both time periods underscore the persistent challenge of incomplete reporting in SRSs.

### 3.4 Trends in therapeutic subgroup reporting

**FIGURE 5:**
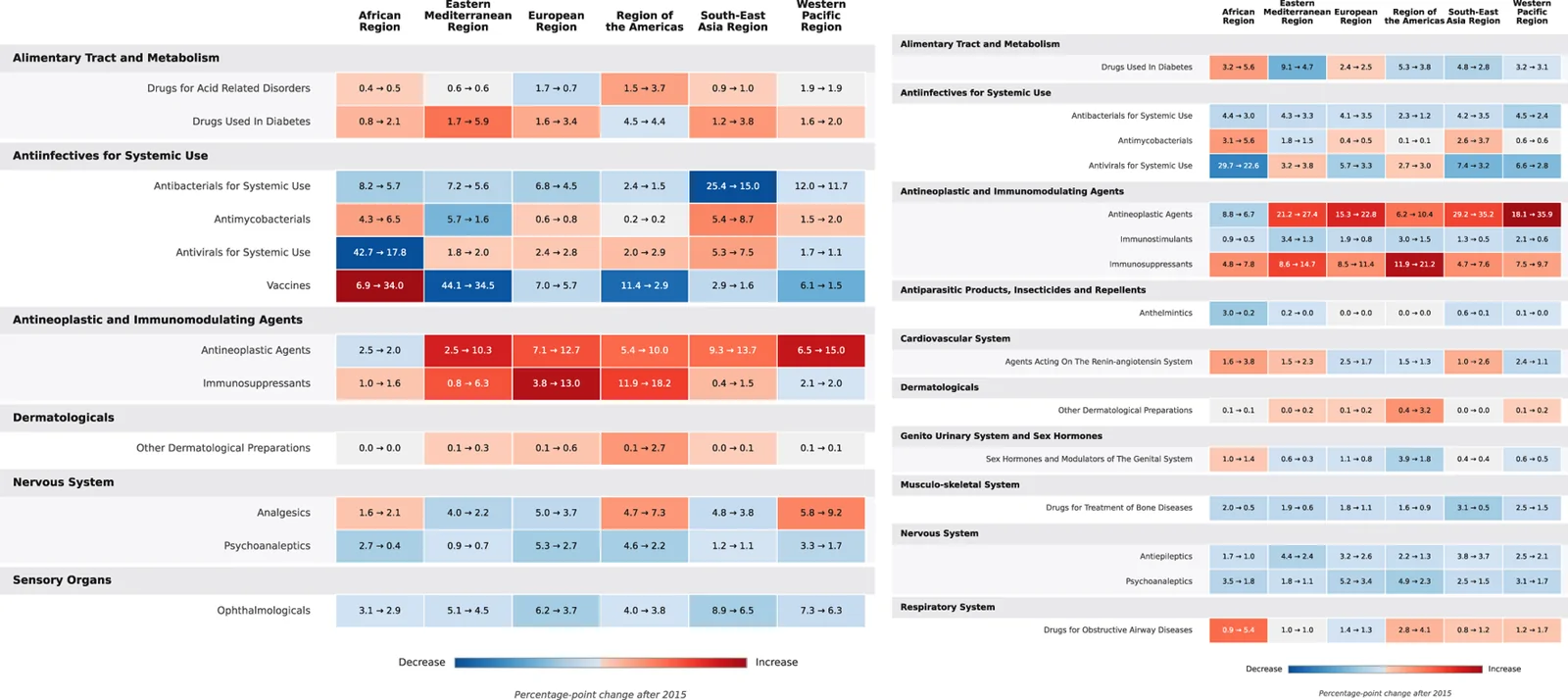
Changes in suspected drug reporting by ATC therapeutic subgroup before and after 1 January 2015, stratified by geographic region. The displayed therapeutic subgroup are the ones that have a change of 2% in at least one region. The reports considered are non-covid.

Shifts in the distribution of reports by suspected medicinal products grouped by ATC therapeutic subgroup are summarised in Figure 5, comparing the periods before and after 1 January 2015, stratified by geographic area across VigiBase, FAERS, and JADER. The same analysis stratified by reporter types is available in Supplementary Figure S8. Since VAERS is essentially a single-product-category database (vaccines), its ATC-level analysis yields no informative variation and it is not discussed further here (CDC, 2026; VAERS - Data, 2026).

Antineoplastic and immunomodulating agents. The most prominent and consistent cross-database trend is the marked increase in reports involving antineoplastic agents and immunosuppressants. In VigiBase, antineoplastic agent reports increase across all regions, from 5.4% to 10.0% in the Region of the Americas, from 7.1% to 12.7% in the European Region, and from 9.3% to 13.7% in South-East Asia Region (Figure 5 top panel). An analogous pattern is observed in FAERS (Figure 5 bottom panel), where reports listing antineoplastic agents rose from 6.2% to 10.4% in the Region of the Americas and from 15.3% to 22.8% in the European Region, with a particularly significant increase in the Western Pacific Region (from 18.1% to 35.9%). The latter is reflected in JADER, with reporting of antineoplastic agent reports increasing from 19.9% to 34.8% (Supplementary Figure S9), consistent with the strong oncology focus of Japanese pharmaceutical development and clinical practice (Nagashima and Furuse, 2022; Nguyen et al., 2023; Matsumura and Mandai, 2026). Immunosuppressants show parallel increases in FAERS (from 11.9% to 21.2% in the Region of the Americas) and VigiBase (from 11.9% to 18.2% in the Region of the Americas and from 3.8% to 13.0% in the European Region). These cross-database trends align well with the global surge in regulatory approvals for oncology and immunomodulatory therapeutics in recent years and are consistent with the pattern described recently for VigiBase (Brand et al., 2026).

The reporter-stratified analysis (Supplementary Figure S8) seems to show that this increase is not confined to a single reporter type. In VigiBase, antineoplastic agent reporting increase among physicians (from 7.5% to 17.1%), consistent with oncology care being predominantly managed by specialists (Horgan et al., 2024). In FAERS, the increase is most pronounced among both consumers (+4.6%) and physicians (+9.0%). In JADER, the increase is observed across all reporter categories, with the largest absolute increase among physicians (from 21.1% to 38.2%).

Anti-infectives for systemic use. Reports involving antivirals for systemic use decline in the African Region, both in VigiBase (from 42.7% to 17.8%) and FAERS (from 29.7% to 22.6%), a trend consistent with the maturation of antiretroviral pharmacovigilance as HIV treatment programmes stabilised (Nachega et al., 2023; Mody et al., 2024). A similar decline is observed in JADER (from 5.2% to 2.4%) and the Western Pacific Region in FAERS (from 6.6% to 2.8%). Antibacterial for systemic use reporting also decreased in VigiBase (−2.5% in the African Region and −10.5% in the South-East Asia Region) and in JADER (−2.7%). In VigiBase, vaccine reports (ATC level 2) show a notable region-specific pattern as they increase dramatically in the African Region (from 6.9% to 34.0%), a pattern that coincides with the growing involvement of national immunisation programmes in the WHO PIDM (Blau, 2023; Brand et al., 2026; Programme for International Drug Monitoring, 2026), while declining in the Eastern Mediterranean Region (from 44.1% to 34.5%), the Region of the Americas (from 11.4% to 2.9%), the European Region (from 7.0% to 5.7%), the Western Pacific Region (from 6.1% to 1.5%), and the South-East Asia Region (from 2.9% to 1.6%).

Nervous system agents. Reports involving nervous system drugs decline in relative share across all three non-vaccine SRSs. Following recent observations (Brand et al., 2026), VigiBase shows a decline in psychoanaleptics reporting, from 5.3% to 2.7% in the European Region and from 4.6% to 2.2% in the Region of the Americas. In FAERS, psychoanaleptic and epileptics decrease in all regions. JADER exhibits similar decreases for both psychoanaleptics and antiepileptics. Antiepileptic reporting drops modestly across all databases and regions. The reporter-stratified analysis (Supplementary Figure S8) shows that these declines are consistent across reporter types, suggesting a genuine proportional shift rather than a reporting artefact.

Other notable patterns. Reports on drugs for treatment of bone diseases decline consistently across VigiBase (Brand et al., 2026) and FAERS in all regions, as do musculoskeletal anti-inflammatory and antirheumatic product reports. Sex hormone reports decline in VigiBase (from 4.3% to 2.7% in consumer reports) and FAERS (from 3.9% to 1.8% in the Region of the Americas). In FAERS, reports on dermatological preparations show a notable increase in the Region of the Americas (from 0.4% to 3.2%). Ophthalmological reports decline modestly across all databases and regions.

### 3.5 Trends in adverse event reporting

**FIGURE 6:**
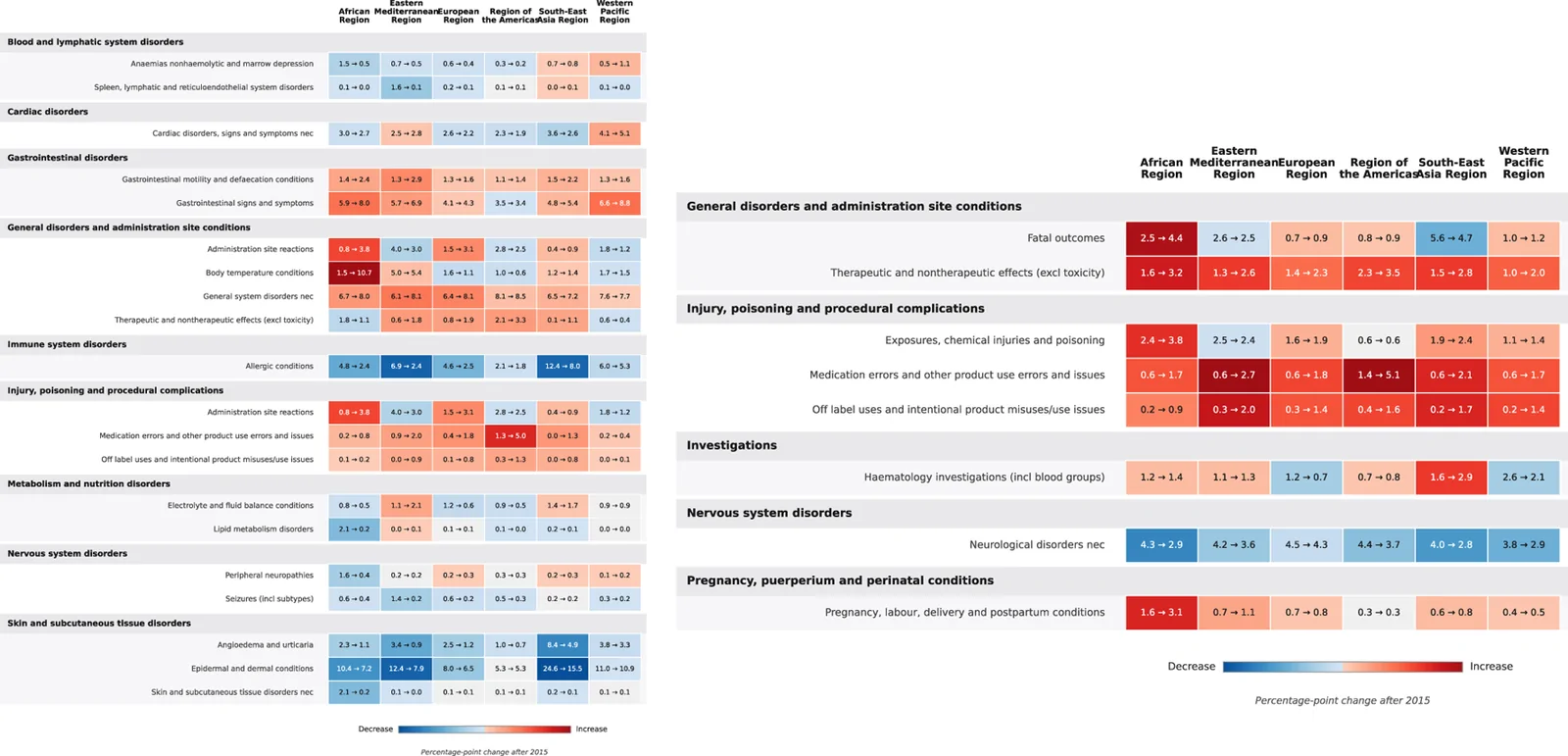
Changes in adverse event reporting by MedDRA™ high level group term (HLGT) before and after 1 January 2015, stratified by geographic region. The displayed HLGTs are the ones that have a change of 1% in at least one region. The reports considered are non-covid.

Shifts in the reporting of adverse events grouped by MedDRA® HLGT are presented in Figure 6, stratified by geographic area across all four databases. The same analysis stratified by reporter type is provided in Supplementary Figure S10.

Traditional adverse drug event categories. As previously reported (Brand et al., 2026), several well-established adverse drug event categories show relative reporting declines, before and after 2015, across databases (Figure 6). In VigiBase, declines are observed in cardiac disorders (−0.4% in the Region of the Americas) and seizures (−0.2% across regions). In FAERS, neurological disorders NEC decline from 4.4% to 3.7% in the Region of the Americas and from 4.5% to 4.3% in the European Region, whereas in VAERS, they decline modestly also in the Region of the Americas (from 5.0% to 4.9%) but markedly in the Western Pacific Region (from 17.8% to 10.2%). Skin and subcutaneous tissue disorders, including angioedema/urticaria and epidermal/dermal conditions, decline in VigiBase across multiple regions, most notably in the South-East Asia Region (epidermal conditions decline from 24.6% to 15.5% and allergic conditions from 12.4% to 8.0%). In JADER, the most notable declines are in hepatic and hepatobiliary disorders (−1.4%), allergic conditions (−2.0%), and epidermal and dermal conditions (−1.7%). These events have historically been recognized as classic adverse drug reactions and even though they’re not losing clinical significance, their relative percentage representation has declined as reports on newer event categories have grown.

General disorders and administration site conditions. Reports of “therapeutic and nontherapeutic effects (excluding toxicity)” increase consistently across regions in both VigiBase and FAERS. In VigiBase, body temperature conditions increase significantly in the African Region (1.5%–10.7%), a pattern consistent with increased reporting from vaccination programmes and the parallel strengthening of immunisation safety surveillance in the region. General system disorders NEC also show modest increases across most databases and regions. Administration site reactions increase in VigiBase, particularly in the African Region (0.8%–3.8%), again consistent with increased vaccine reporting from this region.

Medication errors and product use issues. The most striking cross-database trend is the substantial increase in reports classified under “medication errors and other product use errors and issues.” In FAERS, these reports increase from 1.4% to 5.1% in the Region of the Americas and from 0.6% to 1.7% in the European Region. A similar trend is observed in VigiBase, with increases from 1.3% to 5.0% in the Region of the Americas and from 0.4% to 1.8% in the European Region. Even VAERS, despite its vaccine focus, shows a rise from 0.7% to 5.4% in the Region of the Americas (Supplementary Figure S11). The reporter-stratified analysis (Supplementary Figure S10) confirms that this trend is broad-based, with medication error reports in FAERS increasing among consumers (+3.8%), OHPs (+1.7%), and pharmacists (+3.6%). In VigiBase, the pattern is also observed across all reporter types, with consumers showing the largest absolute increase (+2.7%). This consistency across databases and reporters suggests that the observed increase in reporting is may be associated with the increasing formalisation and visibility of medication error concepts within pharmacovigilance systems and coding practices rather than with a true increase in error frequency (Pera et al., 2024; 2025; Tchijevitch et al., 2025). A similar trend is observed for “off-label uses and intentional product misuses/use issues,” which increase in FAERS (from 0.4% to 1.6% in the Region of the Americas), VigiBase (from 0.3% to 1.3% in the Region of the Americas), and across multiple reporter types in both databases.

Database-specific patterns. VAERS demonstrates some unique patterns reflecting its vaccine-specific scope. Movement disorders (including parkinsonism) show a notable increase in the Western Pacific Region (from 1.0% to 3.8%), as do seizures (from 1.2% to 4.5% in the Western Pacific Region) and sleep disturbances (from 0.0% to 1.6%). Muscle disorders and musculoskeletal disorders NEC also increase in the Western Pacific Region. In JADER, immune disorders NEC increase, as well as vascular haemorrhagic disorders across most reporter types, perhaps owing to the increased anticoagulants use in the ageing Japanese population (Stuby et al., 2024; Sato and Takahashi, 2025).

Reporter-specific trends. The reporter-stratified HLGT analysis (Supplementary Figure S10) reveals some noteworthy reporter-specific patterns. In VigiBase, lawyer-reported cases show striking shifts, with gastrointestinal neoplasms reports increasing by 4.9%, possibly related to litigation trends involving proton pump inhibitors or similar drug classes. In FAERS, lawyer-reported cases also show a dramatic increase in injuries NEC (+1.7%), bone disorders (+2.6%), and gastrointestinal neoplasms (+3.4%).

### 3.6 Signals of disproportionate reporting

To quantify the capacity of each SRS to generate drug safety signals, we computed signals of disproportionate reporting (SDRs) defined as drug-event pairs with a lower bound of the 95% confidence interval of the proportional reporting ratio (PRR05) above 1 and supported by at least five reports (Cutroneo et al., 2024; Slattery et al., 2013). COVID-19 vaccine reports were excluded from this analysis.

As of 31 December 2025, FAERS yields the largest absolute number of SDRs (1,616,441), followed by VigiBase (1,203,070), VAERS (107,390), and JADER (96,174) (Table 1). To compare signal detection efficiency across databases of different sizes and compositions, we defined SDR yield as the ratio of SDRs to the drug-ADE vocabulary (i.e., the number of unique DEPs observed in each database), thereby quantifying the proportion of the observed DEPs space that reaches statistical significance as a disproportionate signal. FAERS exhibits the highest SDR yield (21.1%), followed by VigiBase (16.1%), VAERS (11.0%), and JADER (9.8%). The higher yield in FAERS may reflect its large report volume and structured reporting requirements, which together provide sufficient per-pair reporting depth across a broad drug-event landscape. VigiBase, despite containing the most reports and a comparable vocabulary, shows a lower yield, consistent with its aggregator nature, where heterogeneous reporting practices and potential duplication across contributing national systems may dilute individual drug-event associations. JADER, despite exhibiting the highest reporting entropy (*vide infra*), has the lowest SDR yield, likely because its relatively modest report volume (888,690 reports) is distributed across a comparably sized drug-event vocabulary (984,604 unique pairs), leaving many pairs below the five-report threshold required for SDR detection.

**TABLE 1:** SDR yield and reporting entropy across the four SRSs excluding COVID-19 vaccine reports.

SRS	Deduplicated reports	SDR count	SDR yield	PT vocabulary	PT entropy	Drug-PT vocabulary	Drug-PT entropy
VigiBase	34,012,802	1,203,070	16.13	23,365	0.66	7,458,968	0.81
FAERS	20,205,951	1,616,441	21.07	22,674	0.70	7,672,818	0.82
VAERS	971,016	107,390	10.96	13,338	0.65	979,416	0.77
JADER	888,690	96,174	9.77	10,903	0.71	984,604	0.88

To investigate whether differences in SDR, yield might be related to reporting heterogeneity, we quantified reporting bias using Shannon entropy. We computed the normalised entropy of the distribution of adverse event (PTs) reports across MedDRA™, preferred terms (PT, entropy) and across unique drug-event (DE) pairs (DE, entropy) for each database. A normalised entropy of 1 indicates a perfectly uniform distribution of reports across categories, while lower values indicate greater concentration of reports in fewer categories.

JADER exhibits the highest PT entropy (0.71) and Drug-PT entropy (0.88), suggesting a more uniform distribution of reports across adverse event terms and drug-event combinations despite its smaller vocabulary. By contrast, VAERS shows the lowest ADE entropy (0.65) and Drug-ADE entropy (0.77), indicating that vaccine-related reports are concentrated in fewer adverse event categories, consistent with the narrower product scope of this database. VigiBase and FAERS fall in between, with similar ADE entropy values (0.66 and 0.70, respectively) despite substantial differences in database size (Table 1). Notably, the relationship between reporting entropy and SDR yield is not straightforward under this normalisation. JADER’s high Drug-ADE entropy indicates that reports are distributed most uniformly across its drug-event space, but this very uniformity means that individual drug-event pairs accumulate fewer reports on average, reducing the statistical power to detect disproportionality. Conversely, FAERS achieves the highest SDR yield despite a lower Drug-ADE entropy (0.82), suggesting that a degree of reporting concentration, combined with a large overall report volume, provides the per-pair depth of evidence needed for signal detection. These findings indicate that SDR yield is jointly determined by the breadth of the drug-event vocabulary, the total report volume, and the evenness of reporting across that space, with no single factor dominating.

**FIGURE 7:**
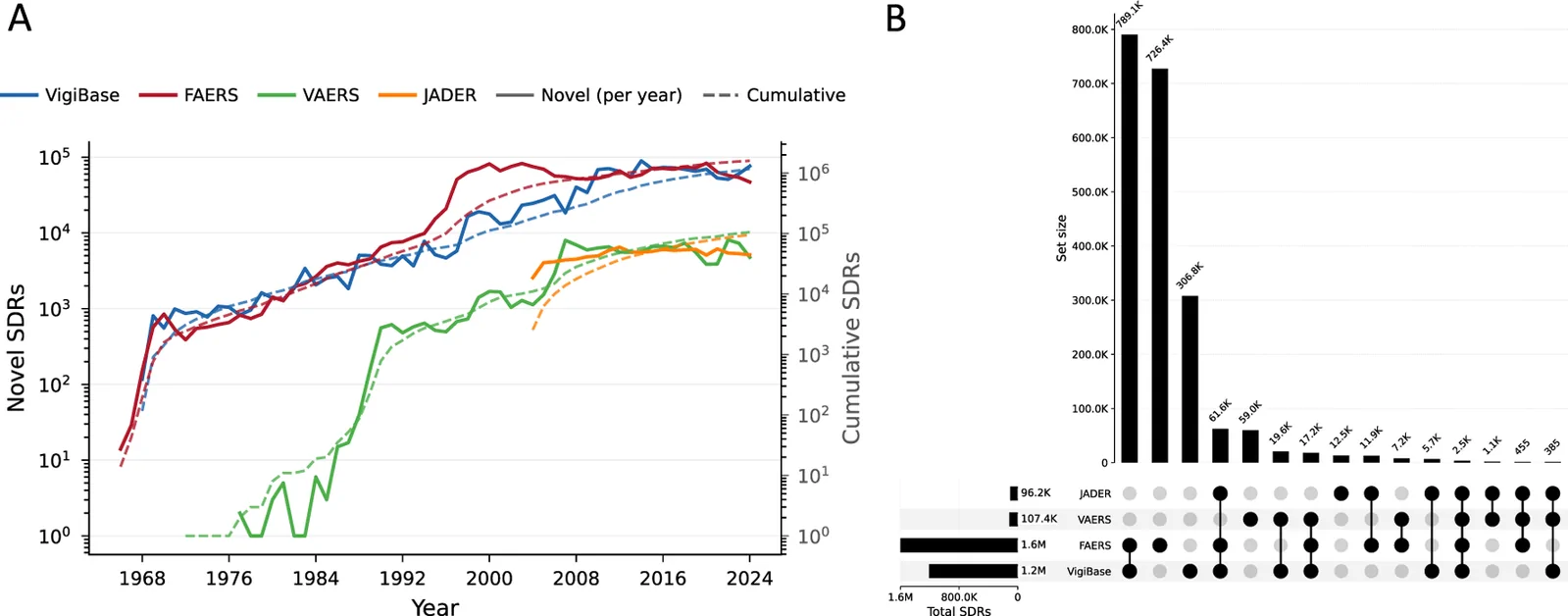
Temporal trends and cross-database overlap of SDRs across the four SRSs, excluding COVID-19 vaccine reports. **(A)** Annual number of novel SDRs (solid lines, left axis) and cumulative SDRs (dashed lines, right axis) by year. **(B)** UpSet intersection plot showing the number of unique drug-adverse event SDRs shared across databases.

The temporal evolution of SDR accumulation mirrors, but does not simply replicate, the growth in report volume (Figure 7A). All four databases show steady accumulation of SDRs, with FAERS and VigiBase reaching approximately 1.6 million and 1.2 million cumulative SDRs by 2024, respectively. The rate of novel SDRs per year, however, reveals divergent trajectories in recent years. In FAERS, the annual number of novel SDRs peaks around 2020 and declines thereafter, suggesting a progressive saturation of the drug-event signal space. Notably, FAERS shows a marked acceleration in novel SDR detection in the early 2000s, coinciding with the 1997 digitalisation of the reporting system (*vide infra*), which increased both the volume and the structured detail of incoming reports.

VigiBase shows a similar plateau around 2014–2016 followed by a modest rebound in 2024. VAERS and JADER generate approximately 5,000–8,000 novel SDRs per year, with VAERS showing greater year-to-year variability, possibly reflecting the episodic nature of vaccine-related reporting. These temporal patterns indicate that while cumulative SDR counts continue to grow, the marginal yield of novel signals per additional year of data is declining in the larger databases.

The overlap of SDRs across databases is presented in Figure 7B. Of the 2,021,522 unique SDRs identified across all four databases, only 2,523 (0.1%) are detected in all four of them, and 79,696 (3.9%) in at least three. By contrast, 1,104,711 SDRs (54.6%) are exclusive of a single database, namely, 726,428 SDRs are only reported in FAERS, 306,806 in VigiBase, 58,961 in VAERS, and 12,516 in JADER. The largest pairwise overlap occurs between VigiBase and FAERS, which share 789,146 SDRs, representing 65.6% of VigiBase’s and 48.8% of FAERS’s total SDR count. This is consistent with the substantial report overlap between these two databases (*vide supra*) and suggesting that shared reports translate into shared signals. JADER has the lowest proportion of unique SDRs, 12,516 (13.0% of JADER’s SDRs), indicating that despite its distinct reporting culture and demographics, most of its signals are also captured by the larger systems. VAERS, being a vaccine-focused database, has the highest proportion of unique SDRs, 58,961 (54.9%), possibly reflecting the detection of vaccine-specific drug-event associations not captured by the other therapeutic modalities. The low proportion of SDRs shared across three or more databases underscores the complementary nature of these systems and supports the value of multi-database signal detection strategies (Montes-Grajales et al., 2023; Crisafulli et al., 2025).

### 3.7 Impact of the 1997 FAERS digitalisation

**FIGURE 8:**
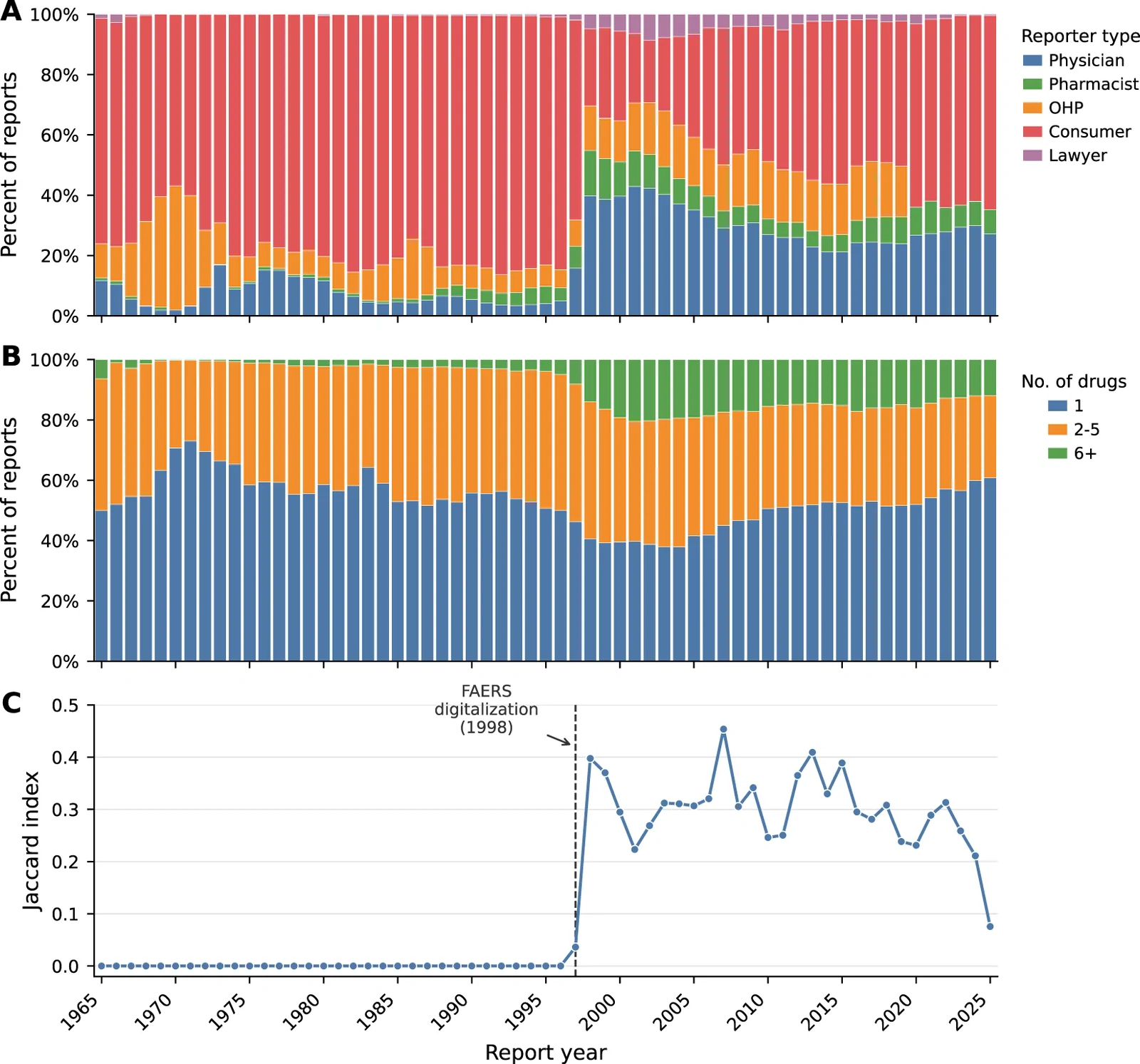
Changes in FAERS reporter composition, polypharmacy reporting, and overlap with VigiBase before and after the 1997 digitalisation. The reports considered are non-covid with non-missing values for each variable. **(A)** Yearly percentage of reports by reporter type. **(B)** Yearly percentage of reports by number of drugs. **(C)** Report overlap between FAERS and VigiBase over report years, measured by Jaccard, with FAERS digitalization marked by a vertical dashed line. The reports considered are with non-missing data for each variable and excluding COVID-19 reports.

In 1997, FAERS (then AERS) underwent a major redesign that enabled structured electronic adverse-event reporting (Mann, 2015; Sonawane et al., 2018). Our analysis identifies two major consequences of this transition in the FAERS data (Figure 8). First, post-digitalisation period is associated with a marked increase in reports from physicians after a pre-digitalisation period dominated by consumer reporting (Figure 8A). This shift may reflect improved integration of electronic reporting tools into clinical workflows, making it easier for physicians to submit reports directly. Second, there is a discernible shift towards increased reporting of polypharmacy, with a growing proportion of reports listing multiple drugs per case following digitalisation (Figure 8B). This may reflect the enhanced capacity of electronic systems to capture complex medication regimens compared to paper-based reporting.

Importantly, the analysis of shared reports between VigiBase and FAERS over time shows that significant report overlap (Section 2.2) between these two databases began after the FAERS digitalisation (Figure 8C). This finding is relevant for researchers conducting analyses using both databases, as it indicates that the shared report population predominantly consists of electronically submitted reports from the post-1997 era and should not pose a concern for historical analyses of FAERS data predating this period.

### 3.8 Strengths and limitations

This study has several notable strengths. To our knowledge, it provides the first comprehensive cross-database comparison of report characteristics and temporal trends across four major SRSs, offering a reference framework for the pharmacovigilance research community. The application of a deduplication algorithm enabled quantification of report overlap between databases, an aspect that has not been sufficiently addressed in previous multi-database studies. The inclusion of JADER alongside the more commonly studied Western databases provides unique insights into reporting patterns in a distinct regulatory and cultural context, particularly highlighting the contrast in reporter types and demographic distributions.

However, there are a few limitations that should be considered. Firstly, there are fundamental differences in the scope, data structure and regulatory context of these SRSs which limits the comparability between some variables. For instance, VAERS does not have comparable reporter type and indication data. Second, as in any pharmacovigilance study, missing data is a limitation. In this case the data completeness for various fields means that the reported cases may not be representative of the events occurring in practice. Finally, all SRSs are subject to known limitations of spontaneous reporting. These include underreporting, stimulated reporting, notoriety bias, and others (Cutroneo et al., 2024; Mestres, 2024; Scholl et al., 2025). Secondly, because analyses were restricted to reports with non-missing values, complete-case analysis may introduce selection bias for variables with high missingness (Supplementary Table S5), where data may be recorded preferentially when clinically relevant. Additionally, databases may apply medical coding with varying specificity and consistency, which can affect comparisons of adverse event profiles. Moreover, spontaneous reporting data do not permit adjustment for confounders such as drug exposure rates, comorbidities, or comedication. Finally, despite deduplication, residual overlap between databases cannot be entirely excluded, which may affect the independence of cross-database comparisons; a formal sensitivity analysis of the deduplication thresholds and of the 1 January 2015 cutoff was not performed. Taken together, these limitations mean that the differences described here should be interpreted as reflecting the characteristics of the reporting systems rather than underlying clinical differences, and that SDR counts reflect database-specific signal-generation behaviour rather than measures of true drug risk.

Analyses of multiple SRSs compound these limitations as different biases may be present in different databases. Temporal influences add an additional layer of complexity, changing reporting formats like in FAERS in 1997, differential impact of global events such as the COVID-19 pandemic, or changing regulatory requirements, all of which can introduce shifts in reporting patterns and potentially signal detection (Montes-Grajales et al., 2023; Brand et al., 2026).

## 4 Conclusion

Here we presented a comparative analysis of four SRSs to determine shared patterns and find notable differences in the trends of spontaneous reporting. Across all systems we found accelerated report growth, increased non-physician contributions and shifts in therapeutic subgroup reporting. On the other hand, we observed differences in demographic profiles, data completeness and COVID-19 vaccination campaigns. This underscores the need of understanding the unique characteristics of each SRS when conducting a multi-SRSs pharmacovigilance study. Additionally, as some of these SRSs pull in reports from others, efforts should be made into deduplication strategies to avoid double-counting. As pharmacovigilance progressively encourages international collaboration and multi-source synthesis, the reference provided in this manuscript could support more informed and nuanced post-marketing safety analysis worldwide.

## Data Availability

The data analyzed in this study is subject to the following licenses/restrictions: FAERS and VAERS are publicly available; VigiBase and JADER require a licence. Requests to access these datasets should be directed to VigiBase: https://who-umc.org/vigibase-data-access/; JADER: https://www.pmda.go.jp/safety/info-services/drugs/adr-info/suspected-adr/0003.html.
