# Supplementary material for: Global patterns and trends in spontaneous reporting systems: VigiBase, FAERS, VAERS, and JADER
Source: Front Drug Saf Regul. 2026 Sep 4;6:1919822. doi: 10.3389/fdsfr.2026.1919822 (PMC13586690; doi:10.3389/fdsfr.2026.1919822)
Supplement: Supplementary file 1 [file Supplementaryfile1.docx]

**Supplementary material for**

**Global Patterns and Trends in Spontaneous Reporting Systems: VigiBase, FAERS, VAERS, and JADER**

Nikola Panajotovikj^1,2^, Jordi Mestres^1,2*^

^1^ Chemotargets SL, Parc Cientific de Barcelona, Barcelona, Spain
^2^ Institut de Quimica Computacional i Catalisi, Universitat de Girona, Girona, Spain

* correspondence to: [jordi.mestres@chemotargets.com](mailto:jordi.mestres@chemotargets.com)

Supplementary Table S1 Percent of report types across VigiBase, FAERS, VAERS, and JADER in non-deduplicated reports.	1

Supplementary Table S2 Percent of report types across VigiBase, FAERS, VAERS, and JADER in deduplicated reports.	2

Supplementary Table S3 Descriptive characteristics of reports in FAERS, VAERS, VigiBase, and JADER after exclusion of COVID-19 vaccine reports.	3

Supplementary Table S4 Temporal comparison of report characteristics before and after 1 January 2015 across FAERS, VAERS, VigiBase, and JADER.	5

Supplementary Table S5 Missingness in non-covid reports across variables and databases.	7

Supplementary Figure S1 Counts of uniquely reported drugs (A) and events (B) in the four SRSs.	8

Supplementary Figure S2 COVID-19 reports in cumulative.	9

Supplementary Figure S3 VigiBase trends in report counts, regions, reporter types, sex, and age.	10

Supplementary Figure S4 FAERS trends in report counts, regions, reporter types, sex, and age.	11

Supplementary Figure S5 VAERS trends in report counts, regions, reporter types, sex, and age.	12

Supplementary Figure S6 JADER trends in report counts, regions, reporter types, sex, and age.	13

Supplementary Figure S7 VigiGrade values over time in VigiBase.	14

Supplementary Figure S8 Shifts in the distribution of reports by suspected medicinal products grouped by ATC therapeutic subgroup, comparing the periods before and after 1 January 2015, stratified by reporter. The displayed therapeutic subgroup are the ones that have a change of 2% in at least one reporter type.	15

Supplementary Figure S9 JADER suspected medicinal products grouped by ATC therapeutic subgroup, comparing the periods before and after 1 January 2015, stratified by geographic area. The displayed therapeutic subgroups are the ones that have a change of 2% in at least one region.	16

Supplementary Figure S10 Reporting of adverse events grouped by MedDRA® HLGTs, comparing the periods before and after 1 January 2015, stratified by reporter. The displayed HLGTs are the ones that have a change of 1% in at least one reporter type.	17

Supplementary Figure S11 VAERS and JADER reporting of adverse events grouped by MedDRA® HLGTs, comparing the periods before and after 1 January 2015, stratified by geographic area. The displayed HLGTs are the ones that have a change of 1% in at least one region.	18

# **Supplementary Table S1** Percent of report types across VigiBase, FAERS, VAERS, and JADER in non-deduplicated reports.

|  | **Report type** | **VigiBase** | **FAERS** | **VAERS** | **JADER** |
| --- | --- | --- | --- | --- | --- |
| Count | Vaccine only | 7 899 768 | 318 951 | 2 440 946 | 51 416 |
|  | Medicine  (regardless of vaccine presence) | 34 498 462 | 26 006 841 | 254 536 | 924 453 |
| Percent | Vaccine only | 18.6% | 1.2% | 90.6% | 5.3% |
|  | Medicine (regardless of vaccine presence) | 81.4% | 98.8% | 9.4% | 94.7% |

# **Supplementary Table S2** Percent of report types across VigiBase, FAERS, VAERS, and JADER in deduplicated reports.

|  | **Report type** | **VigiBase** | **FAERS** | **VAERS** | **JADER** |
| --- | --- | --- | --- | --- | --- |
| Count | Vaccine only | 7 517 144 | 316 579 | 2 055 958 | 49 519 |
|  | Medicine  (regardless of vaccine presence) | 31 977 193 | 19 953 537 | 246 579 | 873 695 |
| Percent | Vaccine only | 19.3 % | 1.6 % | 89.3 % | 5.4 % |
|  | Medicine (regardless of vaccine presence) | 80.7 % | 98.4 % | 10.7 % | 94.6 % |

# **Supplementary Table S3** Descriptive characteristics of reports in FAERS, VAERS, VigiBase, and JADER after exclusion of COVID-19 vaccine reports.

|  |  | **VigiBase** | **FAERS** | **VAERS** | **JADER** |
| --- | --- | --- | --- | --- | --- |
| **Region reported** | **T** | 98.01 | 97.57 | 64.96 | 100 |
|  | **F** | 1.99 | 2.43 | 35.04 | 0 |
| **Region** | **Region of the Americas** | 43.65 | 78.9 | 100 | 0 |
|  | **European Region** | 26.7 | 13.75 | 0 | 0 |
|  | **Western Pacific Region** | 21.08 | 6.24 | 0 | 100 |
|  | **South-East Asia** | 4.06 | 0.53 | 0 | 0 |
|  | **Eastern Mediterranean Region** | 2.94 | 0.33 | 0 | 0 |
|  | **African Region** | 1.57 | 0.25 | 0 | 0 |
| **Reporter reported** | **T** | 65.91 | 86.25 | 0 | 97.66 |
|  | **F** | 34.09 | 13.75 | 100 | 2.34 |
| **Reporter** | **Consumer** | 41.15 | 53.35 |  | 7.37 |
|  | **Physician** | 39.7 | 25.02 |  | 70.53 |
|  | **OHP** | 3.72 | 11.93 |  | 7.35 |
|  | **Pharmacist** | 13.93 | 7.23 |  | 14.73 |
|  | **Lawyer** | 1.49 | 2.46 |  | 0.02 |
| **Age reported** | **T** | 76.57 | 60.4 | 80.16 | 90.84 |
|  | **F** | 23.43 | 39.6 | 19.84 | 9.16 |
| **Age** | **Retirement** | 12.18 | 16.01 | 8.97 | 42.31 |
|  | **Maturity** | 15.79 | 19.33 | 13.58 | 21.63 |
|  | **Midlife** | 32.93 | 35.24 | 26.7 | 20.67 |
|  | **Adulthood** | 29.12 | 23.21 | 28.94 | 8.94 |
|  | **Adolescence** | 2.94 | 2.69 | 5.48 | 2.88 |
|  | **Childhood** | 3.85 | 2.63 | 7.81 | 3.57 |
|  | **Infancy** | 2.98 | 0.75 | 8.5 | 0 |
|  | **Newborn** | 0.22 | 0.14 | 0.02 | 0 |
| **Sex reported** | **T** | 85.81 | 53.81 | 94.68 | 95.69 |
|  | **F** | 14.19 | 46.19 | 5.32 | 4.31 |
| **Sex** | **Male** | 39.63 | 39.68 | 35.19 | 51.14 |
|  | **Female** | 60.37 | 60.32 | 64.81 | 48.86 |
| **Weight reported** | **T** | 5.77 | 21.63 | 0 | 37.21 |
|  | **F** | 94.23 | 78.37 | 100 | 62.79 |
| **Weight** | **Obese** | 33.84 | 28.88 |  | 1.54 |
|  | **Overweight** | 25.6 | 24.59 |  | 5.05 |
|  | **Normal** | 28.96 | 31.63 |  | 44.26 |
|  | **Underweight** | 11.6 | 14.9 |  | 49.15 |
| **Medical outcome reported** | **T** | 27.37 | 60.71 | 8.04 | 72.19 |
|  | **F** | 72.63 | 39.29 | 91.96 | 27.81 |
| **Medical outcome** | **Other** | 48.12 | 50.48 | 0.06 | 79.41 |
|  | **Hospitalisation** | 31.81 | 29.62 | 3.42 | 0 |
|  | **Death** | 10.47 | 11.69 | 22.4 | 8.63 |
|  | **Life threatening** | 4.86 | 3.78 | 27.51 | 0 |
|  | **Disability** | 3.55 | 2.72 | 45.82 | 11.96 |
|  | **Required intervention** | 0.88 | 1.23 | 0 | 0 |
|  | **Congenital anomaly** | 0.31 | 0.48 | 0.8 | 0 |
| **Indication reported** | **T** | 60.2 | 81.91 | 0 | 91.15 |
|  | **F** | 39.8 | 18.09 | 100 | 8.85 |
| **Number of indications in report** | **1** | 82.09 | 75.04 |  | 64.39 |
|  | **2-5** | 16.78 | 23.19 |  | 30.21 |
|  | **6+** | 1.13 | 1.78 |  | 5.4 |
| **Indication reported as "Product used for unknown indication"** | | 17.41 | 27.62 |  | 8.82 |
| **Number of drugs in report** | **1** | 57.01 | 51.72 | 62.27 | 30.46 |
|  | **2-5** | 29.46 | 32.4 | 19.52 | 41.35 |
|  | **6+** | 13.53 | 15.88 | 18.2 | 28.19 |
| **Number of suspected drugs in report** | **1** | 81.17 | 77.42 | 71.9 | 67.02 |
|  | **2-5** | 16.93 | 20.77 | 15.52 | 30.82 |
|  | **6+** | 1.9 | 1.8 | 12.58 | 2.16 |
| **Number of reported adverse events** | **1** | 46.87 | 37.32 | 18.27 | 68.31 |
|  | **2-5** | 45.14 | 50.09 | 55.8 | 29.19 |
|  | **6+** | 7.99 | 12.58 | 25.93 | 2.5 |

# **Supplementary Table S4** Temporal comparison of non-covid report characteristics before and after 1 January 2015 across FAERS, VAERS, VigiBase, and JADER.

|  | | **VigiBase** | | **FAERS** | | **VAERS** | | **JADER** | |
| --- | --- | --- | --- | --- | --- | --- | --- | --- | --- |
|  |  | **Until 2014** | **2015 and after** | **Until 2014** | **2015 and after** | **Until 2014** | **2015 and after** | **Until 2014** | **2015 and after** |
| **Region reported** | **T** | 97.73 | 99.95 | 94.77 | 99.54 | 79.55 | 71.51 | 100.00 | 100.00 |
|  | **F** | 2.27 | 0.05 | 5.23 | 0.46 | 20.45 | 28.49 | 0.00 | 0.00 |
| **Region** | **Region of the Americas** | 55.61 | 42.70 | 79.94 | 78.17 | 100.00 | 100.00 | 0.00 | 0.00 |
|  | **European Region** | 28.43 | 21.41 | 14.01 | 13.57 | 0.00 | 0.00 | 0.00 | 0.00 |
|  | **Western Pacific Region** | 11.70 | 26.40 | 5.37 | 6.85 | 0.00 | 0.00 | 100.00 | 100.00 |
|  | **South-East Asia** | 3.12 | 5.05 | 0.32 | 0.68 | 0.00 | 0.00 | 0.00 | 0.00 |
|  | **African Region** | 0.57 | 1.50 | 0.20 | 0.28 | 0.00 | 0.00 | 0.00 | 0.00 |
|  | **Eastern Mediterranean Region** | 0.57 | 2.94 | 0.16 | 0.45 | 0.00 | 0.00 | 0.00 | 0.00 |
| **Reporter reported** | **T** | 68.86 | 68.06 | 89.76 | 83.78 | 0.00 | 0.00 | 95.12 | 99.04 |
|  | **F** | 31.14 | 31.94 | 10.24 | 16.22 | 100.00 | 100.00 | 4.88 | 0.96 |
| **Reporter** | **Consumer** | 29.72 | 41.55 | 51.04 | 54.94 |  |  | 3.30 | 9.44 |
|  | **Physician** | 53.69 | 36.44 | 24.17 | 25.71 |  |  | 81.99 | 64.91 |
|  | **OHP** | 6.56 | 3.13 | 15.32 | 9.44 |  |  | 3.96 | 8.55 |
|  | **Pharmacist** | 8.27 | 17.26 | 6.00 | 8.19 |  |  | 10.74 | 17.08 |
|  | **Lawyer** | 1.76 | 1.62 | 3.46 | 1.71 |  |  | 0.02 | 0.02 |
| **Age reported** | **T** | 75.71 | 73.76 | 64.28 | 57.69 | 90.17 | 74.95 | 94.93 | 88.28 |
|  | **F** | 24.29 | 26.24 | 35.72 | 42.31 | 9.83 | 25.05 | 5.07 | 11.72 |
| **Age** | **Retirement** | 12.28 | 13.80 | 13.77 | 17.77 | 3.37 | 10.03 | 36.62 | 46.73 |
|  | **Maturity** | 15.02 | 17.80 | 17.08 | 21.07 | 6.50 | 18.27 | 23.44 | 21.08 |
|  | **Midlife** | 32.03 | 33.48 | 36.30 | 34.39 | 14.42 | 22.81 | 22.06 | 19.31 |
|  | **Adulthood** | 27.65 | 24.55 | 26.51 | 20.64 | 22.92 | 16.72 | 10.23 | 7.24 |
|  | **Adolescence** | 3.35 | 2.80 | 2.48 | 2.85 | 8.90 | 7.71 | 3.28 | 2.41 |
|  | **Childhood** | 5.33 | 4.04 | 2.76 | 2.54 | 19.29 | 11.49 | 4.37 | 3.23 |
|  | **Infancy** | 4.10 | 3.28 | 0.90 | 0.63 | 24.53 | 12.97 | 0.00 | 0.00 |
|  | **Newborn** | 0.24 | 0.26 | 0.19 | 0.11 | 0.07 | 0.01 | 0.00 | 0.00 |
| **Sex reported** | **T** | 64.17 | 92.18 | 12.03 | 83.05 | 93.82 | 87.02 | 97.92 | 94.21 |
|  | **F** | 35.83 | 7.82 | 87.97 | 16.95 | 6.18 | 12.98 | 2.08 | 5.79 |
| **Sex** | **Female** | 59.09 | 59.24 | 60.43 | 60.27 | 61.36 | 63.42 | 49.25 | 47.82 |
|  | **Male** | 40.91 | 40.76 | 39.57 | 39.73 | 38.64 | 36.58 | 50.75 | 52.18 |
| **Weight reported** | **T** | 10.06 | 5.21 | 25.53 | 18.82 | 0.00 | 0.00 | 50.00 | 31.41 |
|  | **F** | 89.94 | 94.79 | 74.47 | 81.18 | 100.00 | 100.00 | 50.00 | 68.59 |
| **Weight** | **Obese** | 31.84 | 35.44 | 28.12 | 29.55 |  |  | 1.23 | 1.82 |
|  | **Overweight** | 24.71 | 26.33 | 24.10 | 25.03 |  |  | 4.42 | 5.61 |
|  | **Normal** | 29.17 | 28.81 | 31.02 | 32.23 |  |  | 44.83 | 43.67 |
|  | **Underweight** | 14.28 | 9.42 | 16.76 | 13.19 |  |  | 49.51 | 48.90 |
| **Medical outcome reported** | **T** | 32.07 | 27.45 | 68.82 | 55.01 | 5.86 | 4.56 | 82.04 | 66.73 |
|  | **F** | 67.93 | 72.55 | 31.18 | 44.99 | 94.14 | 95.44 | 17.96 | 33.27 |
| **Medical outcome** | **Other** | 42.07 | 50.44 | 46.99 | 53.40 | 0.30 | 0.09 | 80.77 | 78.48 |
|  | **Hospitalisation** | 35.38 | 31.93 | 30.44 | 28.91 | 11.55 | 3.17 | 0.00 | 0.00 |
|  | **Death** | 11.18 | 10.68 | 11.97 | 11.51 | 18.07 | 20.21 | 7.95 | 9.25 |
|  | **Life threatening** | 5.27 | 4.23 | 4.18 | 3.45 | 29.93 | 27.79 | 0.00 | 0.00 |
|  | **Disability** | 3.46 | 2.14 | 3.55 | 2.01 | 40.08 | 47.70 | 11.28 | 12.27 |
|  | **Required intervention** | 2.21 | 0.32 | 2.30 | 0.33 | 0.00 | 0.00 | 0.00 | 0.00 |
|  | **Congenital anomaly** | 0.42 | 0.27 | 0.58 | 0.39 | 0.07 | 1.05 | 0.00 | 0.00 |
| **Indication reported** | **T** | 51.18 | 68.91 | 68.35 | 91.38 | 0.00 | 0.00 | 90.36 | 91.15 |
|  | **F** | 48.82 | 31.09 | 31.65 | 8.62 | 100.00 | 100.00 | 9.64 | 8.85 |
| **Number of indications in report** | **1** | 81.22 | 81.23 | 76.78 | 74.49 |  |  | 53.81 | 69.33 |
|  | **2-5** | 17.51 | 17.62 | 21.24 | 23.88 |  |  | 37.68 | 26.88 |
|  | **6+** | 1.27 | 1.15 | 1.98 | 1.63 |  |  | 8.50 | 3.79 |
| **Indication reported as "Product used for unknown indication"** | | 11.14 | 21.58 | 15.47 | 33.99 |  |  | 2.04 | 13.27 |
| **Number of drugs in report** | **1** | 46.74 | 54.90 | 48.36 | 54.36 | 25.69 | 28.36 | 21.14 | 33.36 |
|  | **2-5** | 36.80 | 30.27 | 35.12 | 30.29 | 37.63 | 31.72 | 41.09 | 43.00 |
|  | **6+** | 11.94 | 10.21 | 14.02 | 14.54 | 35.30 | 29.13 | 35.99 | 22.04 |
| **Number of suspected drugs in report** | **1** | 79.58 | 78.32 | 79.96 | 75.70 | 28.87 | 43.16 | 65.18 | 66.41 |
|  | **2-5** | 18.22 | 19.48 | 18.87 | 22.06 | 37.70 | 31.04 | 32.27 | 31.54 |
|  | **6+** | 2.20 | 2.20 | 1.17 | 2.24 | 33.43 | 25.81 | 2.55 | 2.04 |
| **Number of reported adverse events** | **1** | 47.18 | 50.18 | 37.73 | 37.11 | 19.02 | 21.83 | 70.16 | 69.59 |
|  | **2-5** | 45.77 | 43.74 | 48.80 | 51.01 | 63.26 | 59.50 | 28.10 | 28.59 |
|  | **6+** | 7.05 | 6.09 | 13.47 | 11.88 | 17.72 | 18.67 | 1.74 | 1.82 |

# Supplementary Table S5 Missingness in non-covid reports across variables and databases.

|  | FAERS | VAERS | VigiBase | JADER |
| --- | --- | --- | --- | --- |
| Reporter | 13.75 | 100 | 34.09 | 2.34 |
| Age | 39.6 | 19.84 | 23.43 | 9.16 |
| Gender | 46.19 | 5.32 | 14.19 | 4.31 |
| Weight | 78.37 | 100 | 94.23 | 62.79 |
| Medical outcome | 39.29 | 91.96 | 72.63 | 27.81 |
| Region | 2.43 | 35.04 | 1.99 | 0 |
| Indication | 18.09 | 100 | 39.8 | 8.85 |

| **A. Drugs**   | **B. Events**   |
| --- | --- |

# **Supplementary Figure S1** Counts of uniquely reported drugs (A) and events (B) in the four SRSs. Drugs were harmonised using a curated drug dictionary, which includes the WHO Drug Dictionary and the ATC classification, and adverse events were matched at the PT level (Methods 2.2).

# **Supplementary Figure S2** R eports in cumulative.

# **Supplementary Figure S3** VigiBase trends in non-covid report counts, regions, reporter types, sex, and age, without missing data.

# **Supplementary Figure S4** FAERS trends in non-covid report counts, regions, reporter types, sex, and age, without missing data.

# **Supplementary Figure S5** VAERS trends in non-covid report counts, regions, reporter types, sex, and age, without missing data.

# **Supplementary Figure S6** JADER trends in non-covid report counts, regions, reporter types, sex, and age, without missing data.

# **Supplementary Figure S7** VigiGrade values over time in VigiBase in non-covid reports.

| VigiBase  | FAERS   |
| --- | --- |
| JADER   | |

# **Supplementary Figure S8** Shifts in the distribution of non-covid reports without missing data by suspected medicinal products grouped by ATC therapeutic subgroup, comparing the periods before and after 1 January 2015, stratified by reporter. The displayed therapeutic subgroups are the ones that have a change of 2% in at least one reporter type.

# **Supplementary Figure S9** JADER suspected medicinal products grouped by ATC therapeutic subgroup, comparing the periods before and after 1 January 2015, stratified by geographic area. The displayed therapeutic subgroups are the ones that have a change of 2% in at least one region. The reports considered are non-covid without missing data.

| VigiBase   | FAERS   |
| --- | --- |
| JADER   | |

# **Supplementary Figure S10** Reporting of adverse events grouped by MedDRA® HLGTs, comparing the periods before and after 1 January 2015, stratified by reporter. The displayed HLGTs are the ones that have a change of 1% in at least one reporter type. The reports considered are non-covid without missing data.

VAERS

JADER

# **Supplementary Figure S11** VAERS and JADER reporting of adverse events grouped by MedDRA® HLGTs, comparing the periods before and after 1 January 2015, stratified by geographic area. The displayed HLGTs are the ones that have a change of 1% in at least one region. The reports considered are non-covid without missing data.
